# The first complete chloroplast genome of *Hylomecon japonica* and its phylogenetic position within Papaveraceae

**DOI:** 10.1080/23802359.2019.1573125

**Published:** 2019-07-12

**Authors:** Yonghua Zhang, Joongku Lee, Xuelian Liu, Zhongshuai Sun

**Affiliations:** a College of Life and Environmental Sciences, Wenzhou University, Wenzhou, China;; b College of Life Science, Tonghua Normal University, Tonghua, China;; c Zhejiang Provincial Key Laboratory of Plant Evolutionary Ecology and Conservation, Taizhou University, Taizhou, China;; d Department of Environment and Forest Resources, Chungnam National University, Daejeon, South Korea

**Keywords:** *Coreanomecon hylomeconoides*, spring ephemeral plant, plastome, phylogenomics

## Abstract

*Hylomecon japonica*, a widespread species in East Asia, is a valuable horticultural and medicinal plant. Here, we obtained the first complete sequence of the *H. japonica* chloroplast genome. The complete cp genome was 160,011 bp long, with a large single-copy region (LSC, 88,165 bp) and a small single copy region (SSC, 18,378 bp) separated by a pair of inverted repeats (IRs, 26,734 bp). The cp genome contained 114 unique genes, including 80 protein-coding genes, 30 tRNA genes, and four rRNA genes. The phylogenetic analysis indicated that *H. japonica* is close related with *Coreanomecon hylomeconoides*.

*Hylomecon japonica* (Thunb.) Prantl et Kündig, the sole species of its genus, is a perennial spring ephemeral plant distributed in China, Japan, Korea and the Russian Far East (Zhang and Christopher 2008; Xu and Wang 2017). It is characterized by bright yellow flowers and various leaf morphology, which has developed as an ornamental plant (Xiao et al. 2013). Meanwhile, as a Chinese folk medicine, it is used for the treatment of arthritis, neuralgia, and eczema (Kim et al. 2003), which contains various active compounds, such as flavonol glycosides and saponins (Lee et al. 2012; Wang 2017). Despite the importance of the species, only one nDNA (NADPH gene) and two *cp*DNA markers (*rpo*B–*trn*C and *trn*G intron regions) have been used for phylogenetic analysis at intraspecific taxonomic level (Xu and Wang 2017), less is known about the chloroplast genome in the genus. In this study, we reported and characterized the complete chloroplast genome of *H. japonica* based on the Illumina paired-end sequencing data. Moreover, the phylogeny of Ranunculales was reconstructed by utilizing the published related species’ chloroplast genome sequences.

Total genomic DNA was extracted from silica-dried leaves collected from Mt. Tianmushan in Zhejiang province, China using a modified CTAB method (Doyle and Doyle 1987). The voucher specimen (*Pan Li*, *LP185533-1*) was collected and deposited in the Herbarium of Zhejiang University (HZU). DNA libraries preparation and pair-end 125 bp read length sequencing were performed on the Illumina HiSeq 2500 platform. About 6.8 Gb of raw data were trimmed and assembled into contigs using CLC Genomics Workbench 8. Then, all the contigs were mapped to the reference cp genome of *Coreanomecon hylomeconoides* Nakai (KT274030; Kim & Kim 2016) using BLAST (NCBI BLAST v2.2.31) search and the draft cp genome of *H. japonica* was constructed by connecting overlapping terminal sequences in Geneious R11 software (Biomatters Ltd., Auckland, New Zealand). Gene annotation was performed via the online program Dual Organellar Genome Annotator (DOGMA; Wyman et al. 2004).

The complete cp genome of *H. japonica* (GenBank accession MK251463) was 160,011 bp long consisting of a pair of inverted repeat regions (IRs with 26,734 bp) divided by two single-copy regions (LSC with 88,165 bp; SSC with 18,378 bp). The overall GC content of the total length, LSC, SSC, and IR regions were 38.8%, 37.4%, 33.2%, and 43.2%, respectively. The cp genome encoded a total of 132 genes, of which 114 were unique and 18 were duplicated in the IR regions. The 114 unique genes contained 80 protein-coding genes, 30 tRNA genes, and 4 rRNA genes.

Maximum likelihood (ML) analyses were performed on a data set that included 80 protein-coding genes for 17 taxa in Ranunculales using RAxML v8.2.10 on CIPRES (http://www.phylo.org) under the GTR + G model. The phylogenetic result (Figure 1) is consistent with the prior phylogenetic study on *Ranunculales* (Wang et al. 2009; Sun et al. 2017). *Hylomecon japonica* exhibited the closest relationship with *Coreanomecon hylomeconoides*.

**Figure 1.**
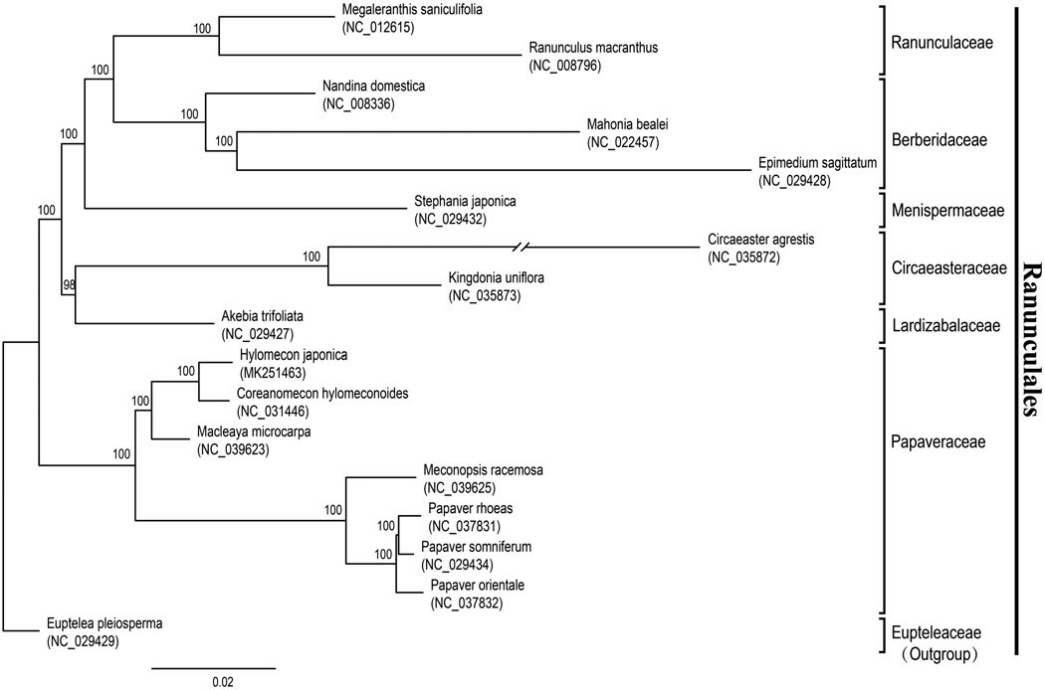
Phylogenetic tree reconstruction of 17 taxa of *Ranunculales* using ML method. Relative branch lengths are indicated. Numbers near the nodes represent ML bootstrap value.
